# A Declining Trend of *Leishmaniasis* Based on Previous Data in Larstan, South of Iran 2007–2017

**DOI:** 10.5334/aogh.1539

**Published:** 2019-03-05

**Authors:** Ahmad Abolghazi, Forogh Ebrahimzadeh, Fatemeh Sharafi, Naser Hatami, Zahra Vafai

**Affiliations:** 1 Department of Medical Parasitology, Jahrom University of Medical Sciences, Jahrom, IR; 2 Department of Medical, Larstan University of Medical Sciences, Larstan, IR; 3 Department of Medical, Shahid behshti University of Medical Sciences, Tehran, IR

## Abstract

**Introduction:**

*Cutaneous Leishmaniasis* is a unicellular of the *Leishmania* type, and 0.7 to 1.2 million people are annually infected by *Cutaneous Leishmaniasis*. Larestan is one of the southern cities of Fars Province. Every year, some issues of *Cutaneous Leishmaniasis* are reported from Larestan. This study aims to analyze the prevalence of *Cutaneous Leishmaniasis* in Larestan from 2007 to 2017.

**Methodology:**

The present study is a cross-sectional descriptive-analytical study which is carried out in Larestan. The study population consists of those people who are infected by *Cutaneous Leishmaniasis* during 2007 to 2017 and are referred to health care centers. The methodology and data collection are done based on the recorded information.

**Results:**

Among 4,965 *Cutaneous Leishmaniasis* infected patients who referred to health care centers of Larestan, 2407 patients (48.47%) are males and 2558 patients (51.53%) are females. In the ten-year time period of the study, 1,315 (26.6%) were infected to *Cutaneous Leishmaniasis* in 2010. The maximum infected group consisted of 1,303 patients ranging from 0–5 years old, and the minimum infected group consisted of 90 patients ranging from 55–60 years old.

**Discussion:**

This study showed that female subjects were more polluted in Larestan city. There is also a significant relationship between age and *cutaneous leishmaniasis*. Finally, it was found that the disease in the city of Larestan has been decreasing.

## 1. Introduction

*Cutaneous Leishmaniasis* is a unicellular of the *Leishmania* type. It is spread by the bite of certain types of *Phlebotomus* sandflies in old world (eastern sphere) and *Lutzomya* in the new world (western sphere). Different clinical forms of this disease cause various kinds of cutaneous symptoms, severe ulcers (mucocutaneous) and fatal symptoms (visceral). The most prevalent type of *Leishmaniasis* is the cutaneous type which is divided into two: dry (urban) and humid (suburban) subtypes. Annually, nearly 0.7 to 1.2 million people worldwide are infected to *visceral Leishmaniasis*. It is estimated that 12–14 million cases of this disease exist all around the world [1,2,3]. According to the analysis, endemic *Leishmaniasis* is reported in more than 98 countries of 5 continents, so that 90% of *visceral Leishmaniasis* is reported from Bangladesh, Brazil, India, Ethiopia, South Soudan, and Soudan; 70–75% of *Cutaneous Leishmaniasis* is reported from Afghanistan, Algeria, Columbia, Brazil, Iran, Syria, Ethiopia, North Sudan, Costa Rica, and Peru [4,5,6]. The prevalence of *Cutaneous Leishmaniasis* in different parts of Iran is fluctuating from 1.8% to 38%; its annual prevalence is 24,630 [5].

This disease has two prevalence types in Iran: visceral (urban) and cutaneous (suburban); in urban *Leishmaniasis* or *Anthroponotic Cutaneous Leishmaniasis*, the cause of disease is *L. Tropica*, its vector is *Phlebotomus Sergenti*, and the main source of infection is human’s body. The urban type of this disease is reported from 14 centers in eight cities all around the country; these reports are mostly from metropolises, such as Tehran, Mashhad, Neyshabour, Shiraz, Kerman, and Bam [4,7].

In the suburban type or *Zoonotic Cutaneous Leishmaniasis*, the main sources of disease are different kinds of rodents, the vector is *Ph. Papatasi*, and the cause of disease is *L. Major. Zoonotic Cutaneous Leishmaniasis* is reported from most of the suburban sections of 17 cities all around the country; these reports are mostly from central Northeast centers (Isfahan, Sarakhs, Lotfabad, Turkmen Sahra, Shahrud, and Varamin Abardej), Western and Southwest centers (Ilam and Khuzestan fields), and Southeast center (Dashtiari District of Balochistan) [8,9,10,11].

*Visceral Leishmaniasis* exists in Mediterranean type in Iran, its cause is *L. Infantum*, and it is reported from all over the country in sporadic form. Unfortunately, this disease is spread in seven districts of country in endemic form [4,9,12].

In Iran, *Leishmania gerbil* and *Leishmania Turanica* are recognized in rodents. However, the pathogenic role of these types is not confirmed in human beings [13].

## 2. Methodology

The present study is a cross-sectional descriptive-analytical study which is carried out in Larestan. Larestan is located in Southern part of Fars Province 915 meters above sea level. Its area is nearly 30,960 km^2^. According to the latest census statistics, this city had a population of 226,879 in 2017 [14]. This city consists of eight towns: Lar, Khur, Latifi, Evaz, Beyram, Banaruiyeh, Juyom, emad Deh, and Sharafuyeh [14] The study population consists of all the *Cutaneous Leishmaniasis* infected patients who referred to health centers from 2008 to 2017. The methodology and data collection are done based on the recorded information of these patients. For the analysis process, gender, duration of infection, age, the infected organ, and living place of the patient are used as main parameters. Then, the collected data are analyzed in SPSS software.

### 2.1. Code of Ethics

All of the ethical provisions are observed in this project.

## 3. Results

**Figure 1:**
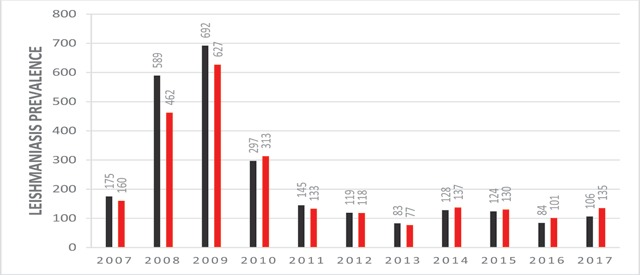
The frequency of males and females with *leishmaniasis* in Larstan, south of Iran from 2007 to 2017.

**Figure 2:**
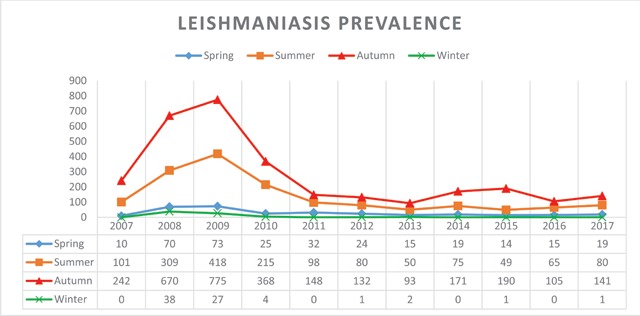
Seasonal trend of *leishmaniasis* in Larstan, south of Iran from 2007 to 2017.

**Figure 3:**
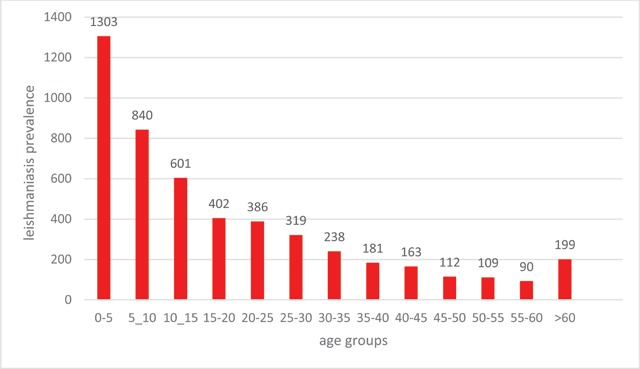
The frequency of males and females with *leishmaniasis* in Larstan, south of Iran from 2007 to 2017.

**Figure 4:**
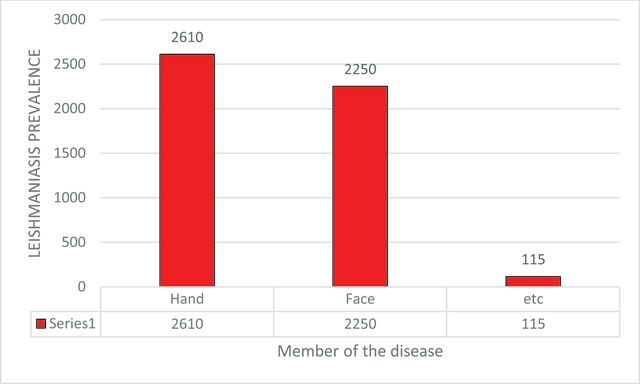
The frequency of member of the *leishmaniasis* in Larstan, south of Iran from 2007 to 2017.

**Figure 5:**
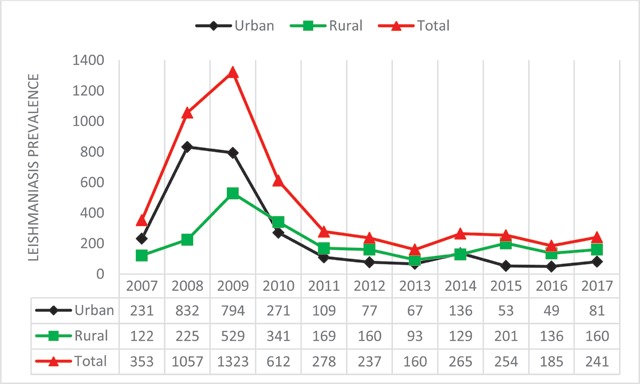
The frequency of people with urban *leishmaniasis* and rural *leishmaniasis* in Larstan, south of Iran from 2007 to 2017.

Among 4,965 *Cutaneous Leishmaniasis* infected patients who referred to health care centers of Larestan, 2,407 patients (48.47%) are males and 2,558 patients (51.53%) are females. This study indicated that more females, in comparison with males, are suffering from *Cutaneous Leishmaniasis* in Larestan. In the diagram of Figure 1, the frequency years of males and females are indicated, respectively. In the ten year time period of the study, maximum infection reports–1,315 (26.06%)–were in autumn in 2010 and the minimum infection reports–160 (3.23%)–were in winter 2013 (Figure 2). The maximum infected group consisted of 1,303 patients ranging from 0–5 years old, and the minimum infected group consisted of 90 patients ranging from 55–60 years old (Figure 3). The most frequently infected organ was the face. After that, hands and feet had the maximum infection reports, which is indicated in the diagram of Figure 4. In the ten-year time period of the study, the number of reported urban *Cutaneous Leishmaniasis* were less than rural *Cutaneous Leishmaniasis* (Figure 5).

## 4. Discussion

The endemic *Leishmaniasis* is reported from Fars Province ant its cities, especially Larestan. A significant number of infections are reported from this city every year [15]. This study aims to analyze the prevalence of *Cutaneous Leishmaniasis* in Larestan. Among the 4,965 patient study population, 2,407 patients (48.47%) are males and 2,558 patients (51.53%) are females. Dehghan et al. from 2008 to 2009, conducted a study in this city and reported that 42.23% males and 57.86% females are infected with this disease. The results of their study are consistent with the results of the present study [16]. Moreover, in the studies of Ayatollahy et al. (2008) and in Abarkuh of Yazd, and Kasiri et al. (2012) in Khorramshahr, it is reported that females are more infected by *Cutaneous Leishmaniasis* than males; these results are consistent with the results of the present study [17]. Rafati et al. (2007–2009) conducted a study in Damghan. They reported that the prevalence of this disease in males is 57.7% and in females is 42.3% [18]. Furthermore, Moghateli et al. (2015) conducted a study in Khash. They reported that the prevalence of this disease is 51% in males and 49% in females. The results of these two studies are in contrast with the results of the present study [19]. It can be said that the possible reason for this contrast is the insignificant relationship between this disease and gender. The year-based results of this study indicated that *Cutaneous Leishmaniasis* in Larestan has a decreasing trend. In the study of Moghateli et al. which was conducted in Khash, the decreasing trend of this disease is reported [19]. These results are consistent with the results of the present study. The possible reason for this decreasing trend can be increased awareness, drought, and increased level of hygiene (health). The results of this study indicated that the range of 0–5 years old makes the maximum infected group, and the range of 55–60 years old makes the minimum infected group. In the study of Moghateli et al. (2013), the range of 0–4 years old made the maximum infected group [19]. Moreover, in the studies of Daloui et al. and Tallari et al. the range of 0–10 years old made the maximum infected group [20,21]. The results of their study are consistent with the results of the present study. The reasons for this high prevalence in some age ranges can be less immunity and more bites of sandflies. The created ulcers of this disease are mostly in the face. It indicates that *Anthroponotic Cutaneous Leishmaniasis* in Larestan is more than *Zoonotic Cutaneous Leishmaniasis*. The results of our study are consistent with the results of the Dehghani et al. (2008) study, which was conducted in Larestan [19].
